# Progress in Vaccination of Prophylactic Human Papillomavirus Vaccine

**DOI:** 10.3389/fimmu.2020.01434

**Published:** 2020-07-10

**Authors:** Xu Zhou, Lihua Sun, Xiaoxiao Yao, Guangquan Li, Yicun Wang, Yang Lin

**Affiliations:** ^1^Department of Gynecology and Obstetrics, The Second Hospital of Jilin University, Changchun, China; ^2^Jilin Provincial Key Laboratory on Molecular and Chemical Genetic, The Second Hospital of Jilin University, Changchun, China

**Keywords:** HPV, vaccine, effectiveness, safety, implementation

## Abstract

The human papillomavirus (HPV) vaccine plays an important role in preventing a series of diseases caused by HPV. Recent studies have shown that as a primary prevention measure, it can considerably prevent HPV infection and HPV-associated cervical cancer. However, studies on the safety, efficacy, and coverage of the HPV vaccine remain insufficient, especially in developing countries. Therefore, in this review, we outlined the recent studies of the HPV vaccine in terms of immunogenicity, safety, efficacy, latest vaccination concepts, and strategies. This review may provide a theoretical basis for use of the HPV vaccine.

## Introduction

One of the malignant tumors with the highest prevalence among women includes cervical cancer. According to the World Health Organization (WHO), there are 570,000 cases and 313,000 deaths added yearly worldwide (1). In the 1880s, German scientist Zur et al. found that correlation between human papillomavirus (HPV) infection and cervical cancer (2). Prophylactic HPV vaccine plays an important role in reducing HPV infection rate, which is a revolutionary step in preventing HPV-related diseases, particularly cervical cancer.

**Figure 1:**
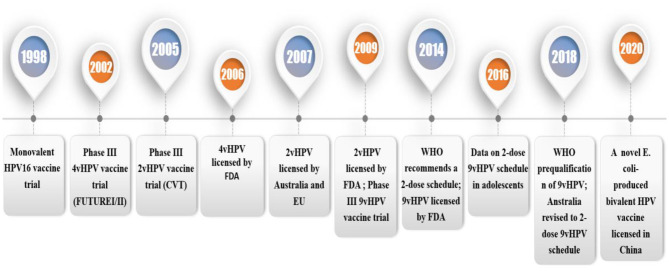
Timeline of trials and licensure/registration of the HPV vaccines. 4vHPV, quadrivalent HPV vaccine; 2vHPV, bivalent HPV vaccine; 9vHPV, non-valent HPV vaccine; FDA, The U.S. Food and Drug Administration; HPV, human papillomavirus; EU, European Union; VLP, virus-like particle.1.1 HPV and HPV infection.

Since 2006, the prophylactic HPV vaccine has been licensed in over 100 countries (3). It is currently available in three types (Table 1): Cervarix (GlaxoSmithKline Biologicals, Belgium), Gardasil (Merck & Co., USA) and Gardasil9 (Merck & Co., USA) (4). Cervarix is a 2-valent HPV (2vHPV) vaccine has two virus-like particles (VLPs) including HPV 16 and 18 VLPs, which causes 70% of cervical malignancies (5). Gardasil is a 4-valent HPV (4vHPV) vaccine containing HPV 16 and 18 VLPs, and type 6 and 11 VLPs, which is associated with 90% of genital wart infections (6). Gardasil 9 is a 9-valent HPV (9vHPV) vaccine for HPV 6/11/16/18/31/33/45/52/58. In 2007, 4vHPV, and 2vHPV were licensed (Figure 1). In 2014, 9vHPV was licensed (7). Currently, vaccination can reduce the incidence of female and male reproductive tract diseases has been determined, including anal and oral HPV infection and cervical, vaginal, vulvar, penile, and anal intraepithelial neoplasia (4). Since the license was granted, HPV infection and incidence rates have dropped considerably worldwide. The safety, efficacy, and duration of prophylactic HPV vaccine have been confirmed by the WHO, which makes vaccination possible to control the occurrence of HPV-associated cervical cancer in humans. This paper reviews the latest progress of three kinds of HPV vaccines and the prospective vaccines. It will provide a theoretical basis for further application of the HPV vaccine.

**Table 1:** Characteristics of HPV vaccines.

**Name**	**Cervarix (2vHPV vaccine)**	**Gardasil (4vHPV vaccine)**	**Gardasil 9 (9vHPV vaccine)**
Antigen	L1 VLP of HPV 16 and 18	L1 VLP of HPV 6, 11, 16, and 18	L1 VLP of HPV 6, 11, 16, 18, 31, 33, 45, 52, and 58
Dose of L1 VLP types	20 and 20 μg	20, 40, 40, and 20 μg	30, 40, 60, 40, 20, 20, 20, 20, and 20 μg
Adjuvant	500 μg alluminium hydroxide,50 μg 3-O-deacylated-4′-monophosphoryl lipid A	225 μg alluminium hydroxyphosphate sulfate	500 μg alluminium hydroxyphosphate sulfate
System	BEVS, baculovirus expression vector system	Yeast	Yeast
Producer cells	Trichoplusia ni (Hi 5) insect cell line infected with L1 recombinant baculovirus	Saccharomyces cerevisiae (baker's yeast) expressing L1	Saccharomyces cerevisiae (baker's yeast) expressing L1
Sodium chloride, mg	4.4	9.56	9.56
L-Histidine, mg		0.78	0.78
Polysorbate 80, μg		50	50
Sodium borate, μg		35	35
Sodium dihydrogen phosphate dihydrate, mg	0.624		
Party	GlaxoSmithKline	Merck	Merck
Approval year	2007: licensure by Australia and EU	2006: licensure by FDA	2014: licensure by FDA
Indications	Females: Cervical precancer and cancer Males: Not approved for use in males	Females: Cervical precancer and cancer; Genital warts Males: Anal precancer and cancer; Genital warts	Females: Cervical precancer and cancer; Genital warts Males: Anal precancer and cancer; Genital warts
Vaccination schedule	0, 1, and 6 months	0, 2, and 6 months	0, 2, and 6 months

*HPV, human papillomavirus; VLP, virus-like particle.2vHPV, 2-valent human papillomavirus; 4vHPV, 4-valent human papillomavirus; 9vHPV, 9-valent human papillomavirus*.

HPV is an epitheliophilic virus belonging to the papillomavirus family and has a variety of hosts of animals and humans (8). On the basis of the ability of HPV to cause lesions, it is divided into two types: high-risk and low-risk types, among which the high-risk type can cause epithelial precancerous lesions and cancer (9). HPV16, 18, 31, 33, 35, 39, 45, 51, 52, 56, 58, 59, and 68 constitute 13 types of high-risk HPV. HPV 16 and HPV 18 have the highest carcinogenicity (5).

HPV may cause cervical, vulvar, vaginal, penile, anal, rectal, and oropharyngeal cancers, which account for more than 80% of HPV-related cancers in the cervix (10). The highest rate of genital HPV infection occurs in women younger than 30 who are sexually active. Eighty to ninety percent of women have at least one HPV infection after sex, although the vast majority are asymptomatic and transient infections that can be cleared within 6–18 months. According to statistics, the HPV infection rate is 30% in the first year after the first instance of sexual behavior and reaches 50% 3 years later (11).

**Figure 2:**
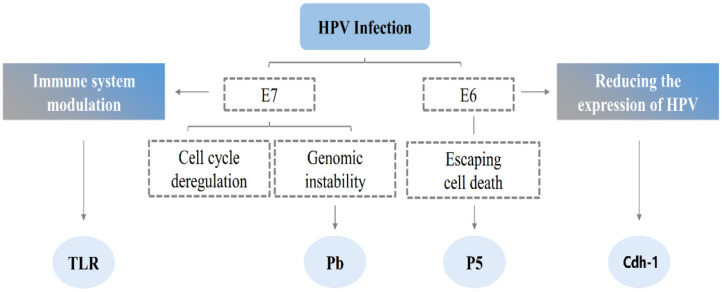
The possible mechanism of HPV infection. Simultaneous molecular targets of the high-risk E6 and E7 oncoprotein. E6 and E7 are known to interact with a diverse range of molecules that are involved in several cellular pathways namely in immune system modulation, invasion, cell cycle deregulation. E6 and E7 share common targets that participate in important processes of cell invasion and inflammation, this cooperation may result in a synergistic mechanism, thereby promoting malignant cell transformation. HPV, human papillomavirus; INF, interferon; TLRs, toll-like receptors; Cdh-1, E-cadherin gene.

The HPV infection is limited to basal epithelial cells, which are infected by micro-scratches on the epithelial surface, which enter the basal cells of the cells with wound healing (12). Thereafter, the DNA of the virus is released from the capsid and enters the nucleus in a free form. In the early stage of infection, since the replication mechanism of the diseased cells has not been effectively expressed, the replicated cells will mature and then die. As the virus continues to infect, it begins to encode E6 and E7 proteins, and E6 degrades the tumor suppressor gene TP53 through the ubiquitin degradation pathway. This blocks the normal growth cycle of cells and prevents cell maturation and apoptosis [(13); Figure 2]. E7 promotes DNA synthesis by interacting with retinoblastoma proteins (14). However, no significant increase in the number of E6 and E7 was observed when the apparent and integrated copies of the viral genome appeared in the same cell (15).

Normal cells transform into cancer cells, due to regulation of the cell cycle by HPV, or due to immune evasion behavior of HPV, which renders the virus undetectable for a long time (16). E6 reduces the expression of HPV antigen by reducing the expression of Cdh-1 on the surface of epithelial cells, whereas E7 prevents TAP1 from activating the immune mechanism of specific cytotoxic T lymphocytes (17). Moreover, E6 and E7 inhibit the transcription of Toll-like receptor 9 and reduce their ability to activate antigen-presenting cells. They also inhibit the synthesis of interferon (18, 19). Finally, the accumulation of genetic alterations and immune evasion causes HPV-induced intraepithelial neoplasia as well as transformation into cancer cells.

### HPV Vaccines

#### Immunogenicity of HPV Vaccines

The cervical location of infection lacks secondary lymphoid tissues. Many memory B cells can reside, prepare antibodies to neutralize the virus (20). However, the levels of antibodies produced by natural infections are not enough to prevent reinfection. The HPV vaccine is immunized by inducing neutralizing antibodies (nAbs) with inactivated or attenuated natural pathogens (21).

The discovery of the pathogenic mechanism of HPV has led to the exploration of its prevention. The prophylactic HPV vaccine is the first one that has been clinically proven to prevent cancer, with landmark significance. In the 1990s, Romanowski et al. found virus-like particles (VLPs) were similar to natural virus particles and did not have the oncovirus genome (22). Studies have shown that in the absence of adjuvants, vaccination with L1VLP vaccines can induce high and long-lasting nAb titers to protect the host from attack by experimental viruses (23). However, the protective effect of L1 VLP on the host is limited by type. To achieve extensive protection, the HPV vaccine needs to contain several key types of L1VLP. The first vaccines were focus on HPV-16 and HPV-18 because of their carcinogenicity. These antibodies are thought to block HPV before entering the basal cell layer that proliferates through the epithelial surface by micro-scratch (24). Once, these antibodies meet HPV, they bind to the virus and prevent it from producing variant cells.

**Figure 3:**
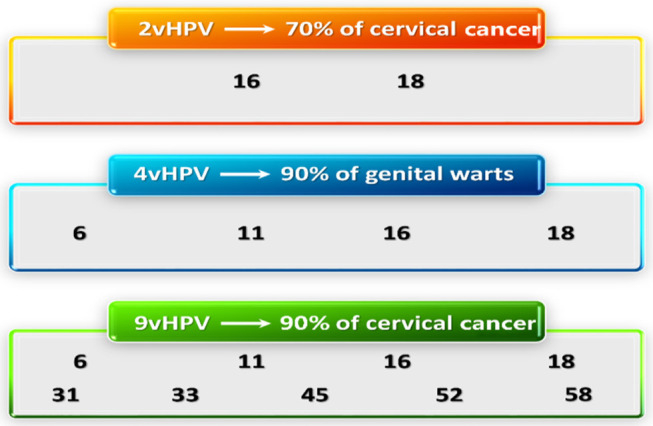
HPV VLP types in VLP vaccines. VLPs in the 2vHPV, 4vHPV, and the 9vHPV are shown with the proportion of neoplastic disease attributed to each group. HPV, human papillomavirus; VLP, virus-like particle.

Several VLP-based vaccines have been approved by many countries around the world. 2vHPV was licensed in 2007 for use and consists of recombinant VLPs from L1 protein of the viral capsid of both HPV types 16 and 18 formulated in AS04. 4vHPV is composed of recombinant VLPs self-assembled from major capsid protein L1 of HPV types 6, 11, 16, 18 adjuvanted with neutral salt aluminum hydroxy phosphate sulfate (25). Merck has increased the 5 types VLP in their vaccine. Compared with 4vHPV, 9vHPV contains more HPV types, and L1 antigen load and adjuvant [(26); Figure 3].

Three vaccines have different immune components (Table 1). 2vHPV and 4vHPV can stimulate the immune system to produce enough HPV16 and HPV18 antibodies. 2vHPV vaccine can promote immunoglobulin G serum response, which is beneficial to the production of T helper cell 1 biased cell response (27, 28), whereas 4vHPV vaccine can produce a T helper cell 2 biased cell response that promotes immunoglobulin G and immunoglobulin A responses (29). Notable, a double-blind randomized controlled trial on 9vHPV reported that among women with HPV infection who have not completed three doses, the vaccine is more than 96% effective against persistent infections caused by HPV 31, 33, 45, 52, and 58 for more than 6 months. After receiving three doses of 9vHPV 5 years, ~77.5–100% women remained seropositive to the types (30). By comparing antibody titers of 9vHPV and 4vHPV, similar antibody responses were observed in the two groups. There was no difference in the incidence of HPV-6, HPV-11, HPV-16, and HPV-18-related diseases among the two groups, indicating the efficacy of 9vHPV for these types was non-inferior to the 4vHPV. Women treated with 9vHPV serum switched to HPV31, 33, 45, 52, and 58 in the seventh month, and the corresponding antibody titers were significantly higher than 4vHPV recipients. When this group was revaccinated again with one dose of 9vHPV, the antibody responses were higher at the first week and the first month after the fourth dose. This indicates that after three doses of the primary series of vaccines, the immune memory of all nine HPV types was affected (7). Compared with 4vHPV and 9vHPV vaccine, the 2vHPV vaccine has higher immunogenicity that elicits more HPV16/18 antibodies and stronger CD4+ T cell responses. Although the 2vHPV vaccine has the lowest antigen concentration among the three vaccines, it contains a late adjuvant AS04 that enhances immunogenicity. It directly persistent antibody responses, stimulates antigen-presenting cells, and elicits significant immune responses (31). Therefore, the 2vHPV vaccine can cause a more intense antibody neutralization reaction. Following vaccination, neutralizing antibodies transude from serum into cervicovaginal secretions where they may provide first-line defense against HPV (32).

The production cost of GlaxoSmithKline is not only more expensive but also more complicated than other system. Other vaccine manufacturers are trying to explore other alternatives (Table 2). It is notable that generating a suitable VLP can reduce the cost of production and the purified cation scheme induces virus particle neutralizing antibodies (33). There are some other HPV vaccines (Table 2). Several other companies are developing L1-based HPV vaccines not only in China but also in India, like Walvax (HPV-16 and HPV-18), China National Biotech Group (HPV-16, HPV-18, HPV-52, and HPV-58) and Health Guard (HPV16, 18, 58), Serum Institute of India (HPV6, 11, 16, 18), etc. (34). For example, Cecolin and Gecolin were developed by purifying L1 VLP from *E. coli*. Adeno associated virus (AAV) (35), bacteriophages (36), or those on a thioredoxin scaffold (37) can improve the immunogenicity of L2 protective epitopes (Table 2).

**Table 2:** Characteristics of other types HPV vaccines.

**System**	**Cecolin**	**Gecolin**	**L1 capsomers**	**RG1-VLP**	**L2-AAV**	**L2 multimer**	**L2-thioredoxin**	**AX03**	**L1-E7 VLP**	**TA-CIN**	**TA-GW**
	***E. coli***	***E. coli***	***E. coli***	**BEVS**	**BEVS or 293T cells**	***E. coli***	***E. coli***	***E. coli***	**BEVS**	***E. coli***	***E. coli***
Antigen	L1 VLP of HPV-16 and HPV-18	L1 VLP of HPV-6 and HPV-11	L1 capsomers of HPV-16	HPV-16 L1-L2 (17–24, 27–38) VLP	L2 peptides of HPV-16 and HPV-31 displayed on AAV VLP	Fusion protein of L2 ~11–88 of HPV-6, HPV-16, HPV-18, HPV-31 and HPV-39	L2 peptide displayed on thioredoxin	L2 peptide displayed on bacteriophage	HPV-16 L1-E7 VLP	HPV-16 L2E7E6 fusion protein	HPV-6 L2E7 fusion protein
VLP types	16 and 18	6 and 11	16	16	16 and 31	6, 16, 18, 31, and 39	/	/	16	16	6
Adjuvant	Alluminium hydroxide	Alluminium hydroxide	/	Alluminium hydroxide	/	Alum	/	/	None	None	AS03
Party	Xiamen Innovax	Xiamen Innovax	R. Garcea, University of Colorado– Boulder	R. Kirnbauer, NCI,Pathovax LLC	2A Pharma	Sanofi, BravoVax	M. Muller, DKFZ	Agilvax, NIAID	Medigene AG	Cantab Pharmaceuticals, Xenova	Cantab Pharmaceuticals, GSK
Status	licensed in china 1n 2020	Phase II	cGMP production	cGMP production	cGMP production	cGMP production	cGMP production	cGMP production	Phase I	Phase II	Phase II

*The first eight vaccines are intended for prevention. The last three vaccines are used for therapeutic use. AAV, adeno-associated virus; AHSS, aluminum hydroxyphosphate sulfate; BEVS, baculovirus expression vector system; cGMP, cyclic GMP; DKFZ, German Cancer Research Center; E. coli, Escherichia coli; GSK, GlaxoSmithKline; GW, genital warts; MPL, monophosphoryl lipid A; NCI, US National Cancer Institute; NIAID, US National Institute of Allergy and Infectious Diseases; TA, tissue antigen*.

#### Cross-Protection of HPV Vaccines

Vaccine-mediated protection is not type-specific, and cross-protection exists after vaccination against a closely related type of the same species. Although 2vHPV is only related to HPV-16 and HPV-18, it has shown 93% effectiveness in preventing CIN3, regardless of HPV type (38, 39). Although the titer of cross-neutralizing antibodies is much lower than that of type-specific antibodies. The latest data confirms that cross-protection can be achieved by reducing the incidence of HPV 31, 33, and 45 exposure within 5 years after receiving the 2vHPV vaccine (40, 41). This may be related to the unique adjuvant system. AS04 can induce inflammatory factors and T cells responses, and inhibit viral transcription or translation (34). These characteristics can be enhanced by exposure to non-vaccine HPV types. It also contributes to cross-protection of humoral and cellular prevention of HPV infection.

Further, 2vHPV can produce antibodies related to HPV-31,−33,−35,−45,−52, and −58 through cross-protection, namely HPVα-7 (related to HPV-18) or α-9 (related to HPV-16) species (42). Regarding the durability of cross-reactions, studies have shown that cross-protection of 2vHPV lasts up to 8 years after vaccination, and there is no evidence that cross-protection of 2vHPV is weakened for women who have received three doses, which may be related to the genomic distance to HPV-16 or −18 (31). Although a single dose of 2vHPV or 4vHPV vaccine can obtain durable type-specific immunity. However, a study also showed that there was no cross-protection after a single 2vHPV or 4vHPV vaccine (43). The 9vHPV vaccine responded to HPV 6, 11, 16 and 18, and was as effective as the 4vHPV vaccine. The 9vHPV only has activity against HPV 6/11/16/18/31/33/45/52/58, it does not prevent diseases caused by other types HPV (44).

#### Adverse Reactions and Safety of HPV Vaccines

Concerns about vaccine safety in some European countries have led to a decline in the acceptance of vaccines. The safety of vaccines has been controversial and has become one of the main obstacles to public vaccination. Vaccination is not completely risk free, and it can also cause short term adverse reactions (AEs). The most common AE found currently are local injection site symptoms, including fatigue, headache, and muscle pain. Compared to 4vHPV vaccine, injection site reactions are more likely to occur in 2vHPV and 9vHPV vaccine (45, 46). Studies have found that in a post-marketing survey conducted on over 1,000 girls vaccinated with 2vHPV, myalgia was the most common systemic event, followed by fatigue and headache. Systemic AEs including fever, nausea, and dizziness have also been frequently reported (47). In 2013, the Japanese government terminated the HPV vaccination program due to the adverse effects of local pain syndrome events and instructed local health authorities not to promote the use of the vaccine until the end of the investigation of its side effects (44). Therefore, the vaccination rate in Japan fell sharply to below 3.9%. The corresponding mortality rate of cervical cancer in women has also risen sharply (46, 48, 49). Some countries also believe that HPV vaccination is associated with the occurrence of orthostatic tachycardia syndrome, whose causes are mostly unclear and difficult to determine (50). An assessment of vaccine safety in Denmark and Sweden, by analyzing 696,000 women who received the 4vHPV vaccine, did not find consistent evidence supporting the causal relationship between vaccines and autoimmune diseases. A study from France showed that vaccination with HPV did not increase the risk of autoimmune outcomes (51). Another analysis of national data in these countries involved 4 million women, among the 800,000 women who received the 4vHPV vaccine, there was no increase in the incidence of multiple sclerosis or demyelinating diseases after vaccination. In Finland, large-scale statistics on vaccinated adolescents have not been found to increase the incidence of autoimmune diseases (52).

Over 15 years of active monitoring and investigation of any safety signals detected by the HPV vaccine, from pre-licensing active and passive monitoring were conducted. Therefore, the HPV vaccine is one of the most well-studied vaccines to date. The Global Vaccine Safety Advisory Committee has been studying safety-related issues of HPV vaccination and has publicly announced the continued safety and benefits of vaccines seven times (53). Moreover, safety data on vaccination from six Asian countries (10 studies, eight on 2vHPV vaccine, and two on 4vHPV vaccine) showed that the HPV vaccine was also safe for Asian populations (54). Many experimental data show that the vaccine does not cause any serious or unexpected adverse events. Therefore, we should restore public confidence in the safety of HPV vaccination by expanding the scope of vaccination and creating awareness about its benefits and safety. This will play a decisive role in the HPV vaccination program and consequently the risk of HPV-associated cancers.

Pregnancy tests were not required before vaccination, and no adverse outcomes occurred among vaccinated pregnant women. However, HPV vaccination during pregnancy is still not recommended. If pregnancy is confirmed after vaccination, the remaining dose should be postponed to the end of pregnancy (41, 55). There are also some reactions during vaccination, although these reactions are caused by the vaccination process, and not by the vaccine itself (56).

#### Immunization Procedure of HPV Vaccines

In the US, the cost of receiving a three-dose 2vHPV or 4vHPV vaccine is between $350 and $500 (57, 58). Reduction of the economic cost of vaccines is particularly important. Research shows that the two-dose of 2vHPV and 4vHPV vaccine injections are not inferior to the three-dose regimen for adolescents (58). A study was initiated in India to research whether less than three doses of vaccine in 6 month could be non-inferior to three doses for girls aged 10–18 years (59).The study shows that the immunogenicity after two doses of HPV vaccination was as effective as three doses. Therefore, two doses are non-inferior to three doses in preventing HPV related diseases in girls aged 15–18 years (59). WHO has proposed two doses of the 2vHPV and 4vHPV vaccines for people under the age of 15 and three for women over the age of 15. However, the two dose effects are very close to that of three doses provided the two doses of the antibody are separated by at least 6 months (58). In recent years, in some countries, the three-dose immunization program has been promoted and licensed to a two-dose immunization program, and two doses of 2vHPV and 4vHPV vaccines have been given to adolescents (9–13 years old or 14 years old) (60). This problem is particularly prominent because of the high cost of the multi-dose regimen and the cumbersome vaccination process, this issue is particularly important. The second dose can be used flexibly, receiving a two-dose instead of a three-dose of the HPV vaccine may help to considerably reduce costs, thereby increasing vaccination coverage. This will potentially protect more girls from cervical cancer in low-income countries (61). An increasing number of trials are trying to demonstrate the effect of one dose of vaccine. In a large case-control from India, women who received a single dose of 4vHPV vaccine were followed for up to 7 years and the result indicates that this vaccine can provide lasting protection against HPV16 and HPV18 related diseases (62). Although inferior to two or three doses, single dose is immunogenic to HPV16 and HPV18 (59). Single-dose vaccination is more affordable, will simplify the logistics of vaccine delivery, save significant vaccination costs, and is particularly beneficial for wider implementation in low and middle-income countries. However, research into the efficacy and immune response of single-dose HPV vaccines may need to be expanded to other target groups, such as boys, alternative age groups, and HIV-positive individuals. All licensed HPV vaccines should be evaluated, and those currently being developed (34). Data about efficiency of a single dose of vaccine are only up to 7 years old. Therefore, the protection provided by single-dose vaccines still requires long-term assessment.

Since April 2016, the European Medicines Agency has approved a two-dose regimen for 9vHPV for teenagers between the ages of 9 and 14, and those who started using the 9vHPV vaccine after the age of 15 or people with immunodeficiency diseases nevertheless recommend a three-dosage regimen (47).

For these three vaccines, people who have received the first vaccine are advised to use the same one to complete the immunization process (63, 64). With the prevalence of the 9vHPV vaccine, there may be uncertainty about completion of the immunization process starting with one 2vHPV or 4vHPV vaccines, and people who have completed immunization with a 2vHPV or 4vHPV vaccine whether need to revaccinate (65). For women who have not completed the three dose immunization of 2vHPV or 4vHPV vaccines (66) need an additional injection of 9vHPV between 6 and 12 months after the second dose of 2vHPV or 4vHPV vaccines, to completely prevent HPV related diseases. However, in order to obtain some additional protection for other types, should be revaccinated with 9vHPV vaccine after 6–12 months, no evidence supports that one single dose vaccine will protect comprehensively (65). For women who have completed three doses of 2vHPV or 4vHPV vaccine, they can receive two doses of 9vHPV vaccine in 6–12 months (67). A randomized trial showed that three doses of a 9vHPV vaccine were given to a girl who had already received the 4vHPV vaccine previously, the HPV 16, 18 titers was two to three times higher than another girl who only receiving 9vHPV vaccine. However, the anti-HPV 31/33/45/52/58 titers were significantly reduced. Moreover, the resulting anti-HPV 31/33/45/52/58 titers are not likely to provide HPV-specific infection protection (68). Given these results and economic cost-effectiveness, it is recommended that a 9vHPV vaccine be used to complete an incomplete vaccination program to expand the scope of protection. For women who have completed three doses of 2vHPV or 4vHPV, it is recommended that a full injection of the 9vHPV 1 year later be completed.

### Progress in Vaccination of HPV Vaccines

**Figure 4:**
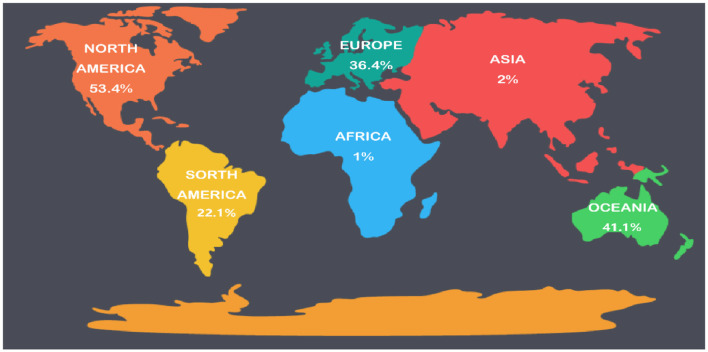
Worldwide HPV vaccination rates. HPV, human papillomavirus.

As a public health issue, cervical cancer can be eliminated by boosting HPV vaccination, cervical cancer screening and related treatments. WHO called for global action to reach this goal in May 2018 (69). A systematic review of the clinical experience of HPV vaccines after use has revealed that its impact in the world is becoming more pronounced, particularly in countries with high vaccination rates. In such countries, the infection of HPV 6/11/16/18 was reduced by more than 90%, the incidence of genital warts caused by HPV decreased by 90%, and the low-grade lesions of the cervix were reduced by 45% (Figure 4). For other sites, published data shows a significant impact of the 9vHPV vaccine on preventing HPV related diseases worldwide (1). However, the process of vaccine introduction has been relatively slow due to the complex production process, excessive costs, the production volume of vaccines cannot meet the demand, the understanding of vaccines is insufficient and the attitude toward vaccination (70).

The predominant factor making vaccines failing to achieve the desired results is insufficient population coverage (71). However, in most highly developed countries, vaccine coverage has not reached effective levels. Crucially, vaccine coverage is <1% in many underdeveloped areas. By October 2014, the vaccination rate was 53.4% in North America, 36.4% in Europe, 41.1% in Oceania, 22.1% in South America, and only 1–2% of 10–20 year old women in Africa and Asia [(70); Figure 4]. Reducing the cost of vaccines (including the need for refrigeration of the vaccine), effective programs and measures adopted by governments are the key factors for long-term success (72). Simultaneously, screening at least one type of cervical cancer for women over 30 years old is an effective way to prevent cervical cancer, although it will increase the government's financial burden. Adolescent women before the sexual debut are considered the best targets for cervical cancer vaccination (41). However, given the long-lasting response to vaccination, it is likely that a children's program will be implemented, such as placing the HPV vaccine in the same bottle as other children's vaccines given under the existing program, to reduce the need for infrastructure. After vaccination of elderly women, the effect is poor, and it has less effect on reducing the incidence of cervical cancer (34). Therefore, vaccination programs targeting specific populations will require further investigation. Furthermore, cost-effective models are still needed to determine the age range and screening interval for HPV vaccination in public health programs (73).

#### Progress in Vaccination of HPV Vaccines in Developed Countries

Since women are the high-risk group of HPV-related diseases, the focus of vaccination programs has always been on women, although in some developed areas vaccination of adolescent men is recommended. Across Europe, HPV vaccination has been implemented in all 28 European Union countries, with vaccination rates as low as 10% in Poland (74) and 43% in Luxembourg (25). The vaccination rate in Italy is 27–83% (26), 86% in the United Kingdom (75), in Belgium Flanders as high as 90% (76). Australia is one of the best to complete the vaccination program and the high efficiency of the 4vHPV vaccine has reduced the infection rate of HPV-6, HPV-11, HPV-16, and HPV-18 in women aged 25 years or older. In male subjects, the prevalence and morbidity of HPV in unvaccinated men was reduced by 70%, and these data suggest that even unvaccinated women can obtain high coverage from vaccines. Australia has changed its cervical cancer screening program to 5 years in 2017 (77). If Australia maintains current HPV vaccination and screening coverage, the annual new cases of cervical cancer may be reduced to <6 cases per 100,000 women by 2020, and to 4 per 100,000 women by 2028 (78). Hence, as a public health issue, Australia might be the first country to eliminate cervical cancer in 20 years (79). A similar situation exists in the Danish survey (80). After the implementation of HPV vaccination program in Britain in 2008, the HPV infection rate showed a decreased trend. Investigations from Scotland also suggest a notable drop in HPV infection and cervical lesions. By analyzing 8854 samples of HPV genotyping, the infection rate of HPV 16/18 decreased from 30.0 to 4.5%. The effective rate of 2vHPV vaccine in women aged 12–13 was 89.1%, and it was confirmed that the HPV type was highly cross-protected in the 2vHPV vaccine test (41).

#### Progress in Vaccination of HPV Vaccines in China

In China, cervical cancer is an important social health issue: there are about 140,000 added cases and more than 34,000 deaths every year (81). This is consistent with trends in socio-cultural norms related to sexual behavior, particularly in young people. The incidence of HPV-related diseases in China increased significantly since 2000, particularly women in their twenties. The trend is largely attributable to lifestyle changes, such as changing sexual behavior (82). The annual incidence of cervical cancer in Chinese urban and rural areas has increased by 9.8 and 15.5%, respectively (83). Therefore, early HPV vaccination in the population can greatly reduce the risk of cervical cancer. Furthermore, it is effective to introduce HPV vaccination timeously for public health in China. The China Food and Drug Administration approved the 2vHPV vaccine in July 2016, which was a groundbreaking decision by the Chinese authorities. Long-term follow-up of clinical studies of the efficacy of 2vHPV vaccine in Chinese women has shown that the effect of the vaccine is consistent with the results of a global study (84). The vaccine is highly immunogenic to Chinese women and does not cause serious adverse reactions. It is effective for HPV 16, 18-related lesions, haptic infections and persistent infections of HPV 31/33/45. In the years following the first injection, the anti-HPV 16/18 antibody response is high and persistent, which is expected to reduce the risk of HPV 16/18 infection in Chinese women. An evaluation of the efficacy and safety of a Chinese woman's 4HPV vaccine for 20–45 years of age indicates that the 4vHPV vaccine has a continuing effect on anal infections and diseases (including high-grade cervical disease) in Chinese women. The time is up to 6.5 years (85). In China, HPV 16/18/58/52/33 are most commonly associated with cervical lesions, suggesting that women infected with HPV 58/52/33 account for a higher proportion of Chinese population than other countries (86). A Chinese bivalent HPV vaccine, including HPV type 16 and 18 VLPs, has been licensed in China in 2020 (Table 1) and completed phase 2–3 trials in terms of efficacy and safety (34). Is has been licensed in China due to the effectiveness and affordability of Chinese HPV vaccine, and the coverage of HPV vaccination in China will increase significantly.

## Conclusion

Public surveillance can promote the effectiveness and safety of HPV vaccines. Furthermore, there are many ways to obtain vaccination information (such as the media) and the information has been misled by misinformation, speculation, and suspicion. This has led to public distrust of the pharmaceutical industry and biomedical technology (87, 88). Moreover, parents and doctors also doubt vaccine safety and have negative attitudes toward it. In addition, parents are concerned that vaccination of HPV vaccine will be examined as acquiescence in sexual activity. The first step to improve the completion rate of HPV vaccine doses for women and men is the training of pediatrics, general practitioners and obstetricians and gynecologists (89). Therefore, we should strive to disseminate vaccine-related science, policies, and recommendations to the non-professional public through various media forms. This will reduce the incidence of cervical cancer in a substantive way (89).

Deaths from oropharyngeal cancer (HPV-related cancers) are projected to increase in 2020, particularly in developed countries (90). Therefore, it is necessary to work toward the full implementation of the vaccination program globally, both for men and women, and to extend the coverage to all medically significant genotypes. In near future, we should also conduct further research on the vaccine itself, optimization of the age of vaccination and the most effective dosage schedule. An increasing number of HPV vaccines are undergoing clinical trials or licensing, such as the Chinese bivalent vaccine, the quadrivalent HPV vaccine in India, and HPV type 6, 11, 16, and 18 VLPs produced by the Indian Serum Institute (Pune, India) (59). Canada has started pairing 9vHPV and 2vHPV (91). There are also studies comparing the effect of one dose of 2vHPV or 9vHPV vaccine with two doses of vaccine (90).These methods can reduce the cost of vaccination and induce a stronger immune response. In conclusion, the latest progress of the HPV vaccine in the immunogenicity, safety, efficacy, latest vaccination concepts, and strategies were reviewed and the prospect solutions were discussed. There are still many challenges in the widespread vaccination of the HPV vaccine. This review provides a theoretical base and applications for the treatment of HPV-related diseases and the further application of the HPV vaccine.

## Author Contributions

XZ, YW, and GL were involved in the study conception and design. LS and XY participated in data acquisition, analysis, and interpretation. XZ and YL wrote the manuscript. All authors have read and approved the manuscript for publication.

## Conflict of Interest

The authors declare that the research was conducted in the absence of any commercial or financial relationships that could be construed as a potential conflict of interest.
